# Deletion of *Yy1* in mouse lung epithelium unveils molecular mechanisms governing pleuropulmonary blastoma pathogenesis

**DOI:** 10.1242/dmm.045989

**Published:** 2020-12-29

**Authors:** Kim Landry-Truchon, Nicolas Houde, Mickaël Lhuillier, Louis Charron, Alice Hadchouel, Christophe Delacourt, William D. Foulkes, Louise Galmiche-Rolland, Lucie Jeannotte

**Affiliations:** 1 Centre de recherche sur le cancer de l'Université Laval, Centre de recherche du CHU de Québec-Université Laval (Oncology Axis), Québec, Canada G1R 3S3; 2 Inserm U1151, Institut Necker-Enfants Malades, Université de Paris, 75743 Paris, Cedex15, France; 3 AP-HP, Hôpital Necker-Enfants Malades, 75743 Paris, Cedex15, France; 4 Department of Medical Genetics, Lady Davis Institute and Segal Cancer Centre, Jewish General Hospital, Montréal, Canada H3T 1E2; 5 Department of Molecular Biology, Medical Biochemistry & Pathology, Université Laval, Québec, Canada G1V 0A6

**Keywords:** YY1, Pleuropulmonary blastoma, Congenital pulmonary airway malformation, miR-125a-3p, EMT, Lung development

## Abstract

Pleuropulmonary blastoma (PPB) is a very rare pediatric lung disease. It can progress from abnormal epithelial cysts to an aggressive sarcoma with poor survival. PPB is difficult to diagnose as it can be confounded with other cystic lung disorders, such as congenital pulmonary airway malformation (CPAM). PPB is associated with mutations in *DICER1* that perturb the microRNA (miRNA) profile in lung. How *DICER1* and miRNAs act during PPB pathogenesis remains unsolved. Lung epithelial deletion of the Yin Yang1 (*Yy1*) gene in mice causes a phenotype mimicking the cystic form of PPB and affects the expression of key regulators of lung development. Similar changes in expression were observed in PPB but not in CPAM lung biopsies, revealing a distinctive PPB molecular signature. Deregulation of molecules promoting epithelial–mesenchymal transition (EMT) was detected in PPB specimens, suggesting that EMT might participate in tumor progression. Changes in miRNA expression also occurred in PPB lung biopsies. miR-125a-3p, a candidate to regulate *YY1* expression and lung branching, was abnormally highly expressed in PPB samples. Together, these findings support the concept that reduced expression of YY1, due to the abnormal miRNA profile resulting from *DICER1* mutations, contributes to PPB development via its impact on the expression of key lung developmental genes.

This article has an associated First Person interview with the joint first authors of the paper.

## Abstract (precis)

**Summary:** YY1 contributes to pleuropulmonary blastoma pathogenesis as a downstream target of abnormal epithelial DICER1-cleaved miRNA profile and as a transcriptional regulator of key players of lung development.

## INTRODUCTION

Pleuropulmonary blastoma (PPB) is a very rare disease with ∼100 new cases diagnosed worldwide each year, although it is the most common pediatric primary lung cancer. Owing to its rarity, PPB is hardly considered by physicians. It arises during fetal life and occurs in young children (Dehner et al., 2015). At the earliest stage, PPB is characterized by low-grade epithelial cystic lesions (Type I), from which septal mesenchyme can overgrow the cystic component into a cystic and solid neoplasm (Type II), and further develops into a purely solid high-grade sarcoma with a poor prognosis (Type III; Hill et al., 2008). It is crucial for the patient that PPB is recognized at the Type I step because it is the most curable stage. Delayed recognition or failure to resect Type I PPB worsens prognosis as cystic PPB can progress over 2-4 years into a solid aggressive tumor. Five-year survival rates range from ∼85% for Type I PPB to ∼45% for Type III (Priest et al., 2006; Messinger et al., 2015). Unfortunately, PPB is a difficult diagnosis to make as symptoms are non-specific and often confounded with those of respiratory infections. Secure diagnosis of PPB relies on histopathologic examination of surgical specimens, but histologic characteristics of Type I PPB are variable. Type I PPB is often mistaken for congenital pulmonary airway malformation (CPAM), another rare but non-tumorous cystic lung disease (Kunisaki et al., 2007; Stocker, 2009).

PPB is the hallmark of *DICER1* syndrome, a pleiotropic tumor-predisposing condition from which family members are at increased risk for developing unusual dysplasias and neoplasms, most of which are associated with perturbed organ development (Dehner et al., 2015). Genetic linkage analysis of familial PPB and related cancers led to the identification of germline loss-of-function mutations in one allele of the *DICER1* gene (Hill et al., 2009; de Kock et al., 2013). *DICER1* encodes an RNaseIII-type enzyme essential for microRNA (miRNA) biosynthesis (Bartel, 2018). In patients with *DICER1* syndrome, the second *DICER1* allele frequently bears a somatic missense mutation in the RNaseIIIb domain of the protein that impairs miRNA biogenesis and modifies the miRNA profile, causing a bias in the generation of miRNAs-3p over miRNAs-5p from their double-stranded precursor (Anglesio et al., 2013; Foulkes et al., 2014; Pugh et al., 2014; Brenneman et al., 2018). The exact role of *DICER1* and miRNAs in PPB development remains unclear, and little is known about the downstream targets of the abnormal epithelial DICER1-cleaved miRNA profile. Overexpression of fibroblast growth factor 9 (FGF9) was documented in cystic lung epithelium from Type I PPB biopsies, a result that correlates with the lung phenotype observed when *Fgf9* is overexpressed in mouse lung epithelium (White et al., 2006; Yin et al., 2015). *Fgf9* lung expression was shown to be under the control of miR-140-5p, providing a potential link between miRNAs and PPB pathogenesis (Yin et al., 2015). However, the broad spectrum of miRNAs affected by the *DICER1* mutation and the complexity of PPB development support the concept that misexpression of several genes must be involved in the disease (Murray et al., 2014; Pugh et al., 2014).

The Yin Yang1 (*Yy1*) gene encodes a ubiquitously expressed zinc-finger-containing transcription factor important for both normal development and cancer (Donohoe et al., 1999; Gordon et al., 2006). We have shown that, in mice, the tissue-specific *Yy1* deletion with the sonic hedgehog (*Shh*)-Cre recombinase, which drives Cre activity in *Shh*-expressing tissues including the respiratory tract epithelium, causes death at birth due to respiratory failure (Harris et al., 2006; Boucherat et al., 2015). It ensues from impaired lung branching and a cystic phenotype evoking that from Type I PPB patients. Furthermore, reduced YY1 protein expression was detected in PPB lung biopsies, whereas no change in expression was observed in CPAM ones. Moreover, *Yy1* lung epithelium-specific mutation affects the expression of several key regulators of lung development in mutant mice. It causes the downregulation of *Shh*, a direct YY1 transcriptional target in lung, and the subsequent upregulation of FGF10 signaling (Boucherat et al., 2015). FGF10 is a major driver of lung bud outgrowth during branching morphogenesis. Its branch-promoting effect is counteracted by SHH, a diffusible factor secreted by the lung epithelium, which signals to the mesenchyme to inhibit FGF10 expression (Swarr and Morrisey, 2015). Thus, the loss of *Yy1* expression in lung epithelium directly impacts *Shh* gene expression, resulting in increased FGF10 signaling that may play a causative role in defective branching and cyst formation. Together, these data led us to speculate that YY1 could be one of the downstream targets of the abnormal epithelial DICER1-cleaved miRNA profile in PPB lung specimens, and that reduced YY1 expression might contribute to PPB pathogenesis by perturbing the expression of key regulators of lung development.

To test the hypothesis, we performed a comparative expression study in lung biopsies from PPB and CPAM patients of genes differentially expressed in *Yy1* mouse mutants, taking advantage of valuable patient cohorts for these two rare lung diseases. We observed changes in gene expression common to lung specimens from both *Yy1* mutant mouse embryos and PPB patients, whereas CPAM samples showed no or little variation in expression. This reveals a distinctive PPB molecular signature that could help with diagnosis of the disease. Moreover, misexpression of genes associated with epithelial–mesenchymal transition (EMT) suggested that this process might contribute to PPB. Specific changes in miRNA expression detected in PPB lung biopsies strengthened the evidence that miRNA misregulation underlies development of the disease. miR-125a-3p, a candidate to regulate *YY1* expression and lung branching, was abnormally highly expressed in PPB lung biopsies. These results support the concept that YY1 is involved in PPB pathogenesis.

## RESULTS

### Distinct gene misregulation in PPB lung biopsies

**Fig. 1.**
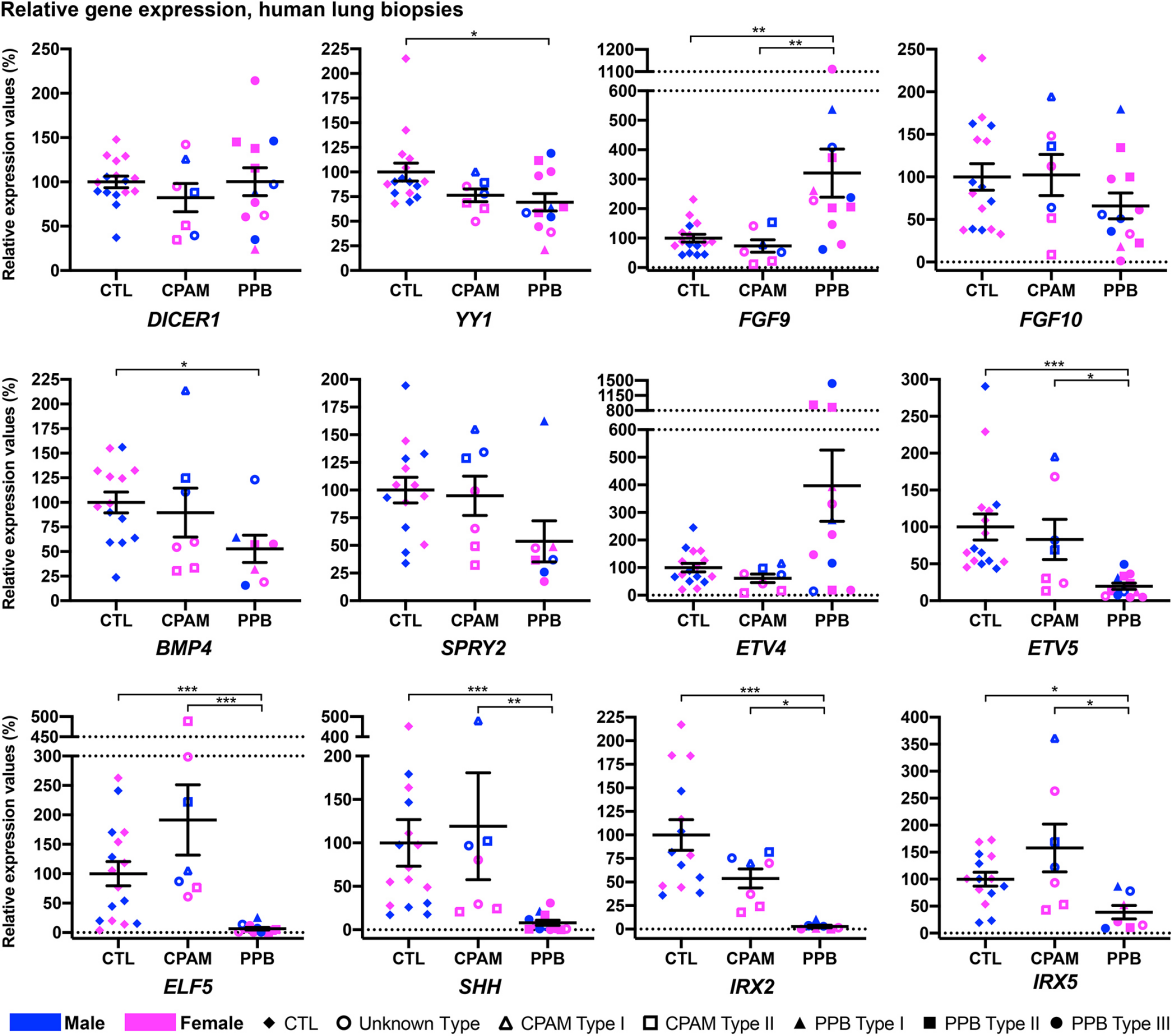
**Molecular distinctions between CPAM and PPB lung biopsies.** qRT-PCR expression analyses of *DICER1*, *YY1*, *FGF9*, *FGF10*, *BMP4*, *SPRY2*, *ETV4*, *ETV5*, *ELF5*, *SHH*, *IRX2* and *IRX5* genes in human lung biopsies from male (blue) and female (pink) control, CPAM (unknown type, Type I and Type II) and PPB (unknown type, Type I, Type II and Type III) patients, indicating a specific PPB molecular signature. Means±s.e.m. are shown. *Padj<0.05, **Padj<0.01, ***Padj<0.001 (Kruskall–Wallis, Dunn's post hoc with Bonferroni correction). CTL, control (*n*=14-16); CPAM, congenital pulmonary airway malformation (*n*=7); PPB, pleuropulmonary blastoma (*n*=7-12).

We previously showed by microarray and quantitative reverse transcription PCR (qRT-PCR) analyses of RNA from lungs at the pseudoglandular stage [embryonic day (E)14.5] of *Yy1*^flox/flox^;*Shh*^+/Cre^ mutant and control mouse embryos that several genes involved in lung development, function and diseases are differentially expressed in *Yy1* mutant specimens (Table S1; Boucherat et al., 2015). These genes included *Fgf10*, *Bmp4*, *Spry2*, *Etv4*, *Etv5*, *Elf5*, *Fgf9*, *Shh*, *Ptch1*, *Hhip*, *Irx2*, *Irx3* and *Irx5*, and their misregulation correlates with the cystic lung phenotype of *Yy1*^flox/flox^;*Shh*^+/Cre^ mutants. To determine whether expression of these genes was similarly modified in pediatric cystic lung diseases, we performed qRT-PCR analyses of total RNA extracted from PPB and CPAM lung biopsies (Fig. 1; Tables S1 and S4). We also examined *DICER1* expression, which was not affected in *Yy1*^flox/flox^;*Shh*^+/Cre^ mutant lungs (Boucherat et al., 2015). *DICER1* transcript levels remained stable in both pathologies. Thus, the *DICER1* mutations occurring in PPB did not affect the expression of the gene. In addition, we measured *YY1* expression because we previously showed reduced YY1 protein expression in PPB (Boucherat et al., 2015). In *Yy1*^flox/flox^;*Shh*^+/Cre^ mutants, *Yy1* expression was specifically abolished in lung epithelium (Boucherat et al., 2015). *YY1* expression was 1.4-times lower in PPB patients than in controls [adjusted *P*-value (*P*adj)=0.05; Fig. 1; Tables S1 and S4].

*Fgf9* lung expression was diminished in *Yy1*^flox/flox^;*Shh*^+/Cre^ mutants (Boucherat et al., 2015). In contrast, in PPB patients, *FGF9* expression was 3.2-times higher than in controls (*P*adj=0.007), whereas no difference was detected between CPAM and control specimens (Fig. 1; Tables S1 and S4). These data were in agreement with the FGF9 overexpression previously reported in PPB lungs (Yin et al., 2015).

We observed that *FGF10* expression in PPB specimens was reduced 1.5-times when compared to controls (*P*adj=0.33), a result contrasting with the significantly elevated *Fgf10* expression described for *Yy1*^flox/flox^;*Shh*^+/Cre^ lung specimens (Fig. 1; Table S4; Boucherat et al., 2015). Decreased FGF10 protein expression was previously reported in PPB patients (Lezmi et al., 2013). Accordingly, when we looked at the expression of FGF10 targets, diminished expression of *BMP4* (1.9-times, *P*adj=0.04) and *SPRY2* (1.9-times, *P*adj=0.08) was seen in PPB samples versus controls. No change was noted for *FGF10*, *BMP4* and *SPRY2* expression in CPAM samples (Fig. 1). Expression of *Etv5*, another FGF10 target, was also significantly reduced in *Yy1*^flox/flox^;*Shh*^+/Cre^ mutants, and a similar observation was made in PPB patients, in whom *ETV5* expression was 5.1-times lower than in controls (*P*adj<0.0001; Fig. 1; Table S4). Moreover, the augmented *Etv4* expression seen in *Yy1*^flox/flox^;*Shh*^+/Cre^ mutants was reproduced in PPB lung biopsies, in which a 4.0-times increase in expression of *ETV4* was observed (*P*adj=0.32; Fig. 1). ETV4 is a bona fide mediator of FGF10 signaling in lungs (Herriges et al., 2015). In CPAM patients, *ETV4* and *ETV5* RNA levels were similar to those of controls (Fig. 1). We also looked at *ELF5* expression, an FGF-sensitive transcription factor in lung, which showed a significantly decreased expression in *Yy1*^flox/flox^;*Shh*^+/Cre^ mutants (Boucherat et al., 2015). In PPB patients, *ELF5* expression was 15.4-times lower than in controls (*P*adj=0.0005), whereas in CPAM samples, *ELF5* expression was 1.9-times higher than in controls (*P*adj=0.92; Fig. 1; Tables S1 and S4; Metzger et al., 2007).

YY1 positively regulates *Shh* epithelial expression by direct binding to upstream regulatory sequences (Boucherat et al., 2015). *SHH* was 12.6-times less expressed in PPB lung samples versus controls (*P*adj<0.0001), and showed no major changes in CPAM specimens. This correlated with the decreased SHH protein expression previously observed in PPB patients (Fig. 1; Lezmi et al., 2013). Finally, we looked at the expression of *IRX2* and *IRX5*, two established transcriptional regulators of lung branching morphogenesis, showing diminished expression in *Yy1*^flox/flox^;*Shh*^+/Cre^ lung specimens (van Tuyl et al., 2006). A significant reduction in RNA levels, 36.1-times for IRX2 (*P*adj=0.0001) and 2.6-times for IRX5 (*P*adj=0.03), was observed in PPB patients compared to controls, as in *Yy1* mouse mutants. In CPAM samples, *IRX2* expression was slightly reduced compared to controls (1.9-times, *P*adj=0.65), whereas an opposite trend was observed for *IRX5* (1.6-times increase compared to controls, *P*adj>0.99; Fig. 1; Table S4). Taken together, the data indicate that PPB exhibits a distinctive molecular signature when compared to CPAM, which includes the ligands FGF9 and SHH as well as the lung epithelial transcriptional regulators ETV4, ETV5, ELF5, IRX2 and IRX5. Except for the FGFs, this molecular signature closely matches that of the *Yy1* lung epithelium loss-of-function mouse model.

### Involvement of FGFR2 isoform switching in PPB

**Fig. 2.**
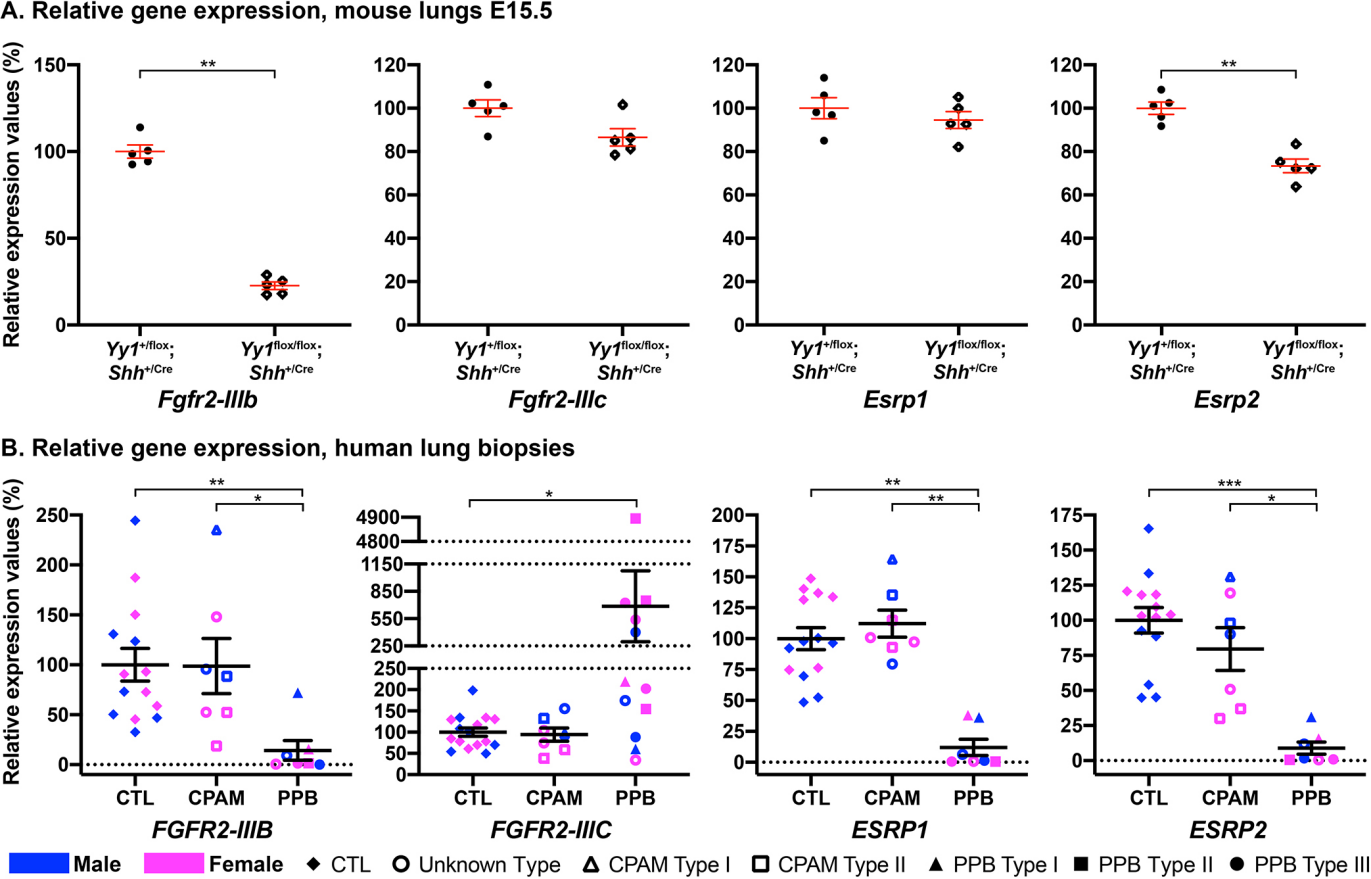
**FGFR2 isoform switching in PPB lung biopsies.** (A) qRT-PCR analyses of *Fgfr2-IIIb*, *Fgfr2-IIIc*, *Esrp1* and *Esrp2* expression levels in lungs from E15.5 controls and *Yy1*^flox/flox^;*Shh*^+/Cre^ mouse embryos (*n*=5 per genotype). **P<0.01 (Mann–Whitney). (B) qRT-PCR analyses of *FGFR2-IIIB*, *FGFR2-IIIC*, *ESRP1* and *ESRP2* expression levels in human lung biopsies from male (blue) and female (pink) control, CPAM (unknown type, Type I and Type II) and PPB (unknown type, Type I, Type II and Type III) patients, indicating a severe downregulation of *ESRP1* and *ESRP2*, which likely explains the FGFR2 isoform switching in PPB lung biopsies. Means±s.e.m. are shown. *Padj<0.05, **Padj<0.01, ***Padj<0.001 (Kruskal–Wallis, Dunn's post hoc with Bonferroni correction). CTL (*n*=14-16), CPAM (*n*=7), PPB (*n*=7-12).

The lack of significant variation in *FGF10* expression in PPB patients, combined with the differences in RNA levels of the FGF10 pathway effectors *ETV4*, *ETV5* and *ELF5*, raised questions about the role of FGF10 in PPB pathogenesis. In lung, FGF10 signals via the fibroblast growth factor receptor 2 IIIB (FGFR2-IIIB). Accordingly, both *Fgf10* and *Fgfr2-IIIb* null mutant mouse embryos present a similar lung agenesis phenotype (Sekine et al., 1999; De Moerlooze et al., 2000). Therefore, we looked at *FGFR2-IIIB* expression. In *Yy1*^flox/flox^;*Shh*^+/Cre^ lung specimens, *Fgfr2-IIIb* RNA levels were decreased 4.4-times (*P*=0.008; Fig. 2A; Tables S1 and S4). Similarly, *FGFR2-IIIB* showed 7.0-times lower expression in PPB specimens than in controls (*P*adj=0.003), while no change was observed in CPAM samples (Fig. 2B). These data were in agreement with the decrease in FGFR2-IIIB protein expression levels previously reported in PPB lung biopsies (Lezmi et al., 2013).

The *FGFR2* gene encodes two major isoforms generated by alternative splicing. FGFR2-IIIB is mainly expressed in lung epithelium, and FGFR2-IIIC is primarily expressed in mesenchyme, but has also been described in epithelial cancer cell progression (Ranieri et al., 2016). Unlike FGFR2-IIIB, FGFR2-IIIC does not bind FGF10, but binds FGF9 (Ornitz and Itoh, 2015). In PPB specimens, *FGFR2-IIIC* was 6.9-times more expressed than in controls (*P*adj=0.03), whereas the levels in CPAM samples remained similar to those in controls (Fig. 2B). No significant variation in *Fgfr2-IIIc* RNA levels was observed in *Yy1*^flox/flox^;*Shh*^+/Cre^ lung specimens (Fig. 2A).

The inverse variation in *FGFR2-IIIB* and *FGFR2-IIIC* expression in PPB specimens suggested the deregulation of alternative splicing and variant switching. *FGFR2* splicing was shown to be under the control of ESRP1 and ESRP2, two epithelial cell-type splicing RNA-binding proteins. Ectopic expression of either protein favors the FGFR2-IIIB epithelial isoform, while loss of expression of ESRP1 and ESRP2 proteins causes a switch towards the FGFR2-IIIC mesenchymal variant (Warzecha et al., 2009). qRT-PCR assays revealed that both *ESRP1* and *ESRP2* genes showed significantly decreased expression in PPB lung specimens, by 8.3-times (*P*adj=0.002) and 11.2-times (*P*adj=0.0003), respectively, versus controls, whereas no variation was detected in CPAM samples (Fig. 2B). In mice, the loss of *Yy1* function in lung epithelium did not perturb *Esrp1* expression but negatively affected *Esrp2* (Fig. 2A). Thus, reduced *ESRP1* and *ESRP2* expression provides a likely explanation for the FGFR2 isoform shift in PPB.

### Expression of EMT markers in PPB

**Fig. 3.**
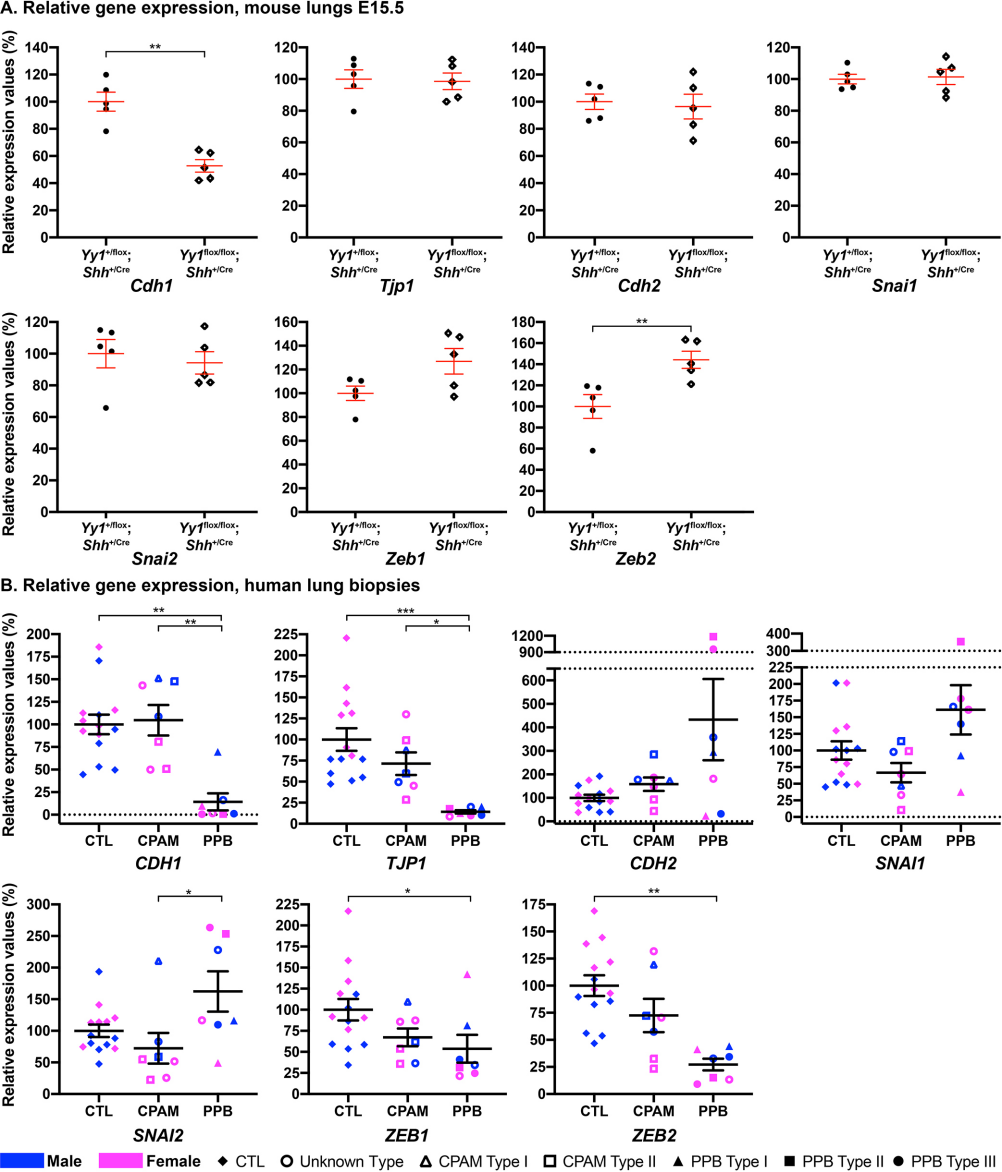
**Expression of epithelial–mesenchymal transition (EMT) markers in PPB lung biopsies.** (A) qRT-PCR expression analyses of *Cdh1*, *Tjp1*, *Cdh2*, *Snai1*, *Snai2*, *Zeb1* and *Zeb2* genes in lungs from E15.5 controls and *Yy1*^flox/flox^;*Shh*^+/Cre^ mouse embryos (*n*=5 per genotype). **P<0.01 (Mann–Whitney). (B) qRT-PCR analyses of *CDH1*, *TJP1*, *CDH2*, *SNAI1*, *SNAI2*, *ZEB1* and *ZEB2* genes in human lung biopsies from male (blue) and female (pink) control, CPAM (unknown type, Type I and Type II) and PPB (unknown type, Type I, Type II and Type III) patients, suggesting that EMT is promoted in PPB specimens. Means±s.e.m. are shown. *Padj<0.05, **Padj<0.01, ***Padj<0.001 (Kruskal–Wallis, Dunn's post hoc with Bonferroni correction). CTL (*n*=14), CPAM (*n*=7), PPB (*n*=7).

**Fig. 4.**
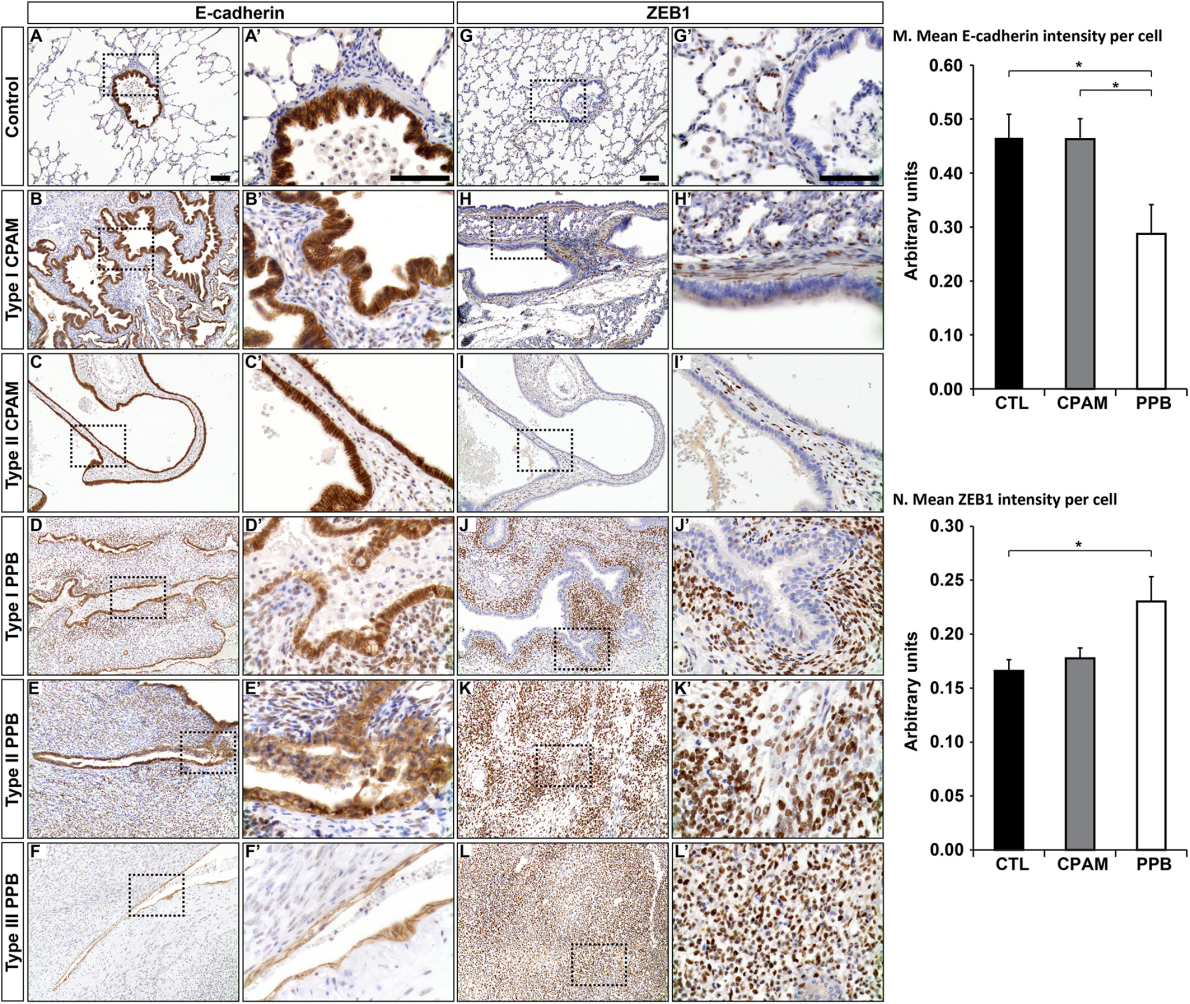
**E-cadherin and ZEB1 protein expression levels are affected in PPB lung biopsies.** (A-L′**)** Representative micrographs showing E-cadherin (A-F′) and ZEB1 (G-L′) immunostaining in human lung biopsies from control (A,A′,G,G′), CPAM Type I and Type II (B-C′,H-I′) and PPB Type I, Type II and Type III (D-F′,J-L′) patients, showing reduced E-cadherin and augmented ZEB1 protein expression in PPB lung specimens. Right columns show higher magnification images of the boxed areas from the left columns. (M,N) Quantification of E-cadherin (M) and ZEB1 (N) staining intensity in epithelium was achieved with QuPath software. Means±s.e.m. are shown. *P<0.05 (unpaired Student's *t*-test). CTL (*n*=6-7), CPAM (*n*=6), PPB (*n*=5-6). Scale bars: 100 µm.

The FGFR2-IIIB to FGFR2-IIIC isoform switch is associated with EMT and malignant progression (Zhao et al., 2013; Ranieri et al., 2016). To assess whether the FGFR2 variant shift seen in PPB was related to EMT, we looked at the expression of key molecular players of EMT (Fig. 3; Table S1). *CDH1*, which encodes the epithelial protein E-cadherin, showed 7.0-times lower expression in PPB biopsies than in controls (*P*adj=0.002), whereas RNA levels in CPAM samples remained similar to those in controls. *Cdh1* also showed 1.9-times lower expression in *Yy1*^flox/flox^;*Shh*^+/Cre^ lung specimens than in control specimens (*P*=0.008). In agreement with the qRT-PCR data, lower E-cadherin protein expression was detected in PPB lung epithelium sections than in control sections by immunohistochemistry (IHC). Quantification of E-cadherin staining intensity using the QuPath software indicated that E-cadherin protein expression was 1.6-times lower in PPB specimens than in controls (*P*=0.03; Fig. 4A-F,M; Bankead et al., 2017). RNA levels of *TJP1*, encoding the tight junction protein ZO-1, were also 7.1-times lower in PPB specimens than in controls (*P*adj=0.0002; Fig. 3B; Table S4). Reduced expression of the *CDH1* and *TJP1* epithelial markers indicated a loss of the epithelial phenotype in PPB specimens. This observation was combined with a non-significant increase (4.3-times) in the expression of *CDH2*, encoding the N-cadherin mesenchymal marker (*P*adj=0.17), and a 1.6-times increase in the expression of *SNAI1* and *SNAI2* genes (*P*adj=0.51 and 0.42, respectively)*.* These two genes code for the evolutionarily conserved transcription factors SNAI1 and SNAI2/SLUG, which repress E-cadherin expression and promote EMT and mesenchymal identity (Fig. 3B; Table S4; Lamouille et al., 2014). No variation in *TJP1*, *CDH2*, *SNAI1* and *SNAI2* expression was detected in CPAM patients (Fig. 3B) or in *Yy1*^flox/flox^;*Shh*^+/Cre^ lung specimens (Fig. 3A).

SNAI transcription factors activate the expression of ZEB genes, which encode two zinc-finger E-box-binding homeobox factors, ZEB1 and ZEB2, which suppress E-cadherin expression (Guaita et al., 2002). However, *ZEB1* and *ZEB2* RNA levels were reduced 1.9-times (*P*adj=0.03) and 3.7-times (*P*adj=0.0006), respectively, in PPB patients, suggesting that expression of other factors important for ZEB transcriptional control might be perturbed (Zhang et al., 2019). In CPAM biopsies, *ZEB1* and *ZEB2* RNA levels presented a non-significant decrease, by 1.5-times (*P*adj=0.55) and 1.4-times (*P*adj=0.51), respectively (Fig. 3B; Tables S1 and S4). ZEB gene expression was also shown to be tightly controlled at the post-transcriptional level by miRNAs (Cano and Nieto, 2008). We therefore investigated ZEB1 protein expression by IHC. Increased ZEB1 expression was detected in PPB specimens compared to controls and CPAM sections (Fig. 4G-L). Quantification of ZEB1 staining intensity with the QuPath software showed that ZEB1 protein was 1.4-times more expressed in PPB specimens than in controls (*P*=0.03; Fig. 4N; Table S4).

**Fig. 5.**
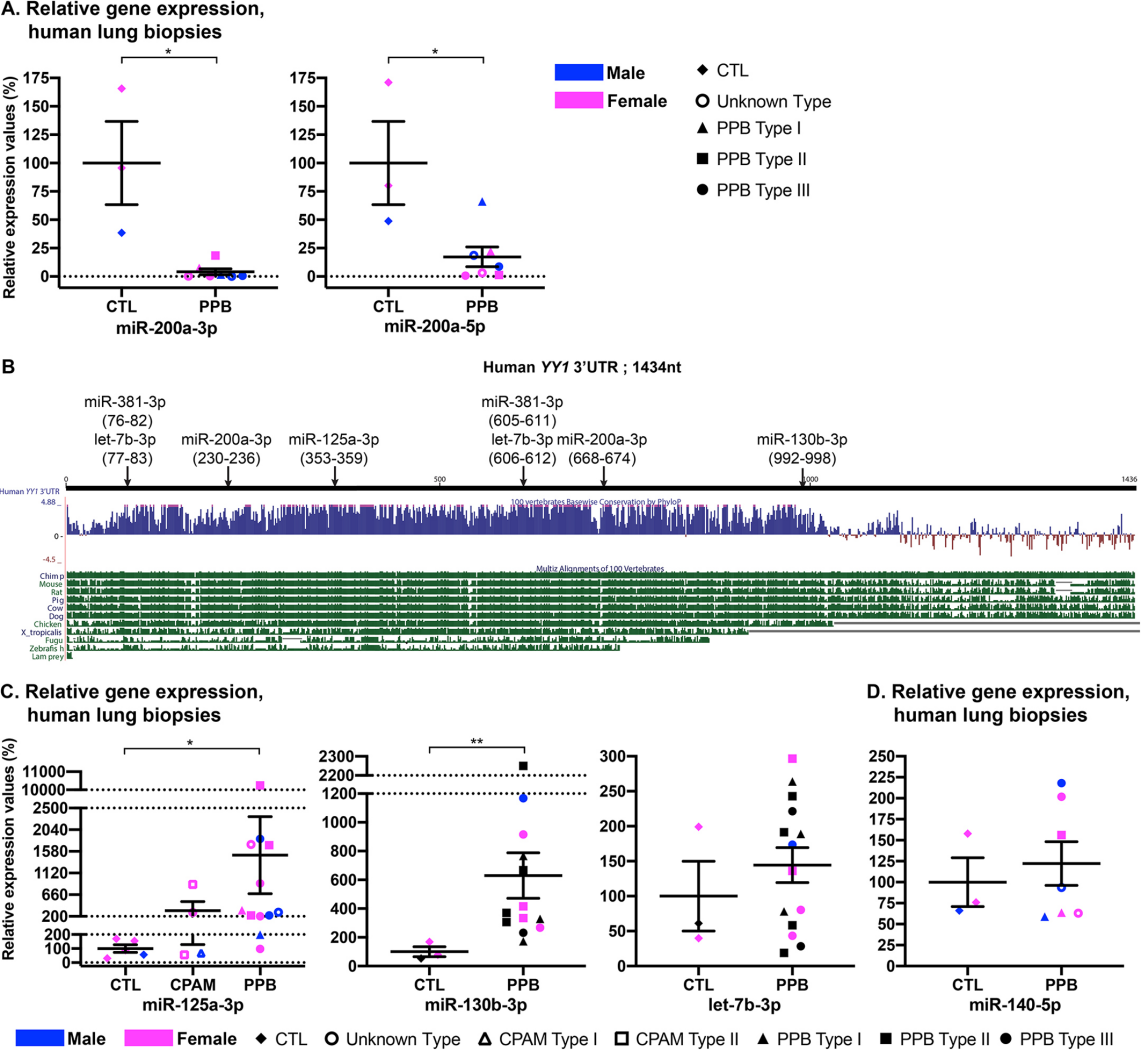
**Misregulation of miRNA expression in PPB lung biopsies.** (A) Expression of miR-200a-3p and miR-200a-5p was assessed by qRT-PCR Taqman miRNA assays in human lung biopsies from male (blue) and female (pink) control and PPB (unknown type, Type I, Type II and Type III) patients. (B) Comparison of human *YY1* 3′UTR sequence to other species using the University of California Santa Cruz (UCSC) genome browser (https://genome.ucsc.edu/) revealed high conservation. Arrows indicate the predicted binding locations of miR-125a-3p, let-7b-3p, miR-130-3p, miR-381-3p and miR-200a-3p in the human *YY1* 3′UTR sequence. Positions are in parentheses. (C,D) Expression of miR-125a-3p, miR-130b-3p, let-7b-3p (C), and miR-140-5p (D) was assessed by qRT-PCR Taqman miRNA assay in human lung biopsies from male (blue) and female (pink) control and PPB (unknown type, Type I, Type II and Type III) patients. miR-125a-3p expression was also tested in CPAM (unknown type, Type I and Type II) specimens. Means±s.e.m. are shown. *P<0.05, **P<0.01 (Mann–Whitney for miR-200a-3p, miR-200a-5p, miR-130b-3p, let-7b-3p and miR-140-5p) and *Padj<0.05 (Kruskal–Wallis, Dunn's post hoc with Bonferroni correction for miR-125a-3p). CTL (*n*=3-5), CPAM (*n*=4), PPB (*n*=7-14).

Members of the miR-200 family control EMT by downregulating the expression of ZEB factors at the post-transcriptional level (Gregory et al., 2008). A double-inhibitory feedback loop exists, and ZEB transcription factors were shown to directly suppress miR-200 expression, which allows maintenance of ZEB expression and promotes EMT (Bracken et al., 2008; Burk et al., 2008). Therefore, increased ZEB1 protein expression detected in PPB samples raised questions about miR-200 expression in patient lung biopsies. This was assessed by measuring miR-200a-5p and miR-200a-3p expression levels using a qRT-PCR Taqman miRNA assay. We found a significant reduction of both miRNAs in PPB specimens, which might underlie the augmented ZEB1 protein expression (Fig. 5A; Table S4). Thus, FGFR2 isoform switching combined with changes in expression of genes and miRNAs associated with EMT suggested that a number of mechanisms converge to promote EMT during PPB pathogenesis.

### Misregulation of miRNA production in PPB lung specimens

miRNAs derive from either the -3p or -5p hairpin strand of their double-stranded precursor (pre-miRNA). They negatively modulate gene expression post-transcriptionally via base pairing, generally in the 3′UTR of target mRNAs to direct their degradation and/or inhibit their translation (Bartel, 2018). The *DICER1* RNaseIIIb missense mutations found in PPB patients are known to modify the miRNA profile by causing a bias in the generation of miRNAs-3p over miRNAs-5p (Anglesio et al., 2013; Pugh et al., 2014). This was also confirmed by miRNA serum profiling of a PPB patient with a mutated DICER1 RNaseIIIb domain (Murray et al., 2014).

*YY1* transcript levels in PPB patients were similar to those measured in CPAM patients (Fig. 1). However, YY1 protein expression was reduced in PPB lung biopsies but not in CPAM patients (Boucherat et al., 2015). No *YY1* mutations were found by exome sequencing in PPB patients, indicating that reduced YY1 protein expression was not caused by mutations in *YY1* coding sequences (Pugh et al., 2014). Together, these data suggested that reduced YY1 protein expression in PPB patients might result from defective post-transcriptional regulation. Several miRNAs have been shown to target the 3′UTR of *YY1*, and, in many cases, they contribute to tumorigenesis (Yang et al., 2013; Zhang et al., 2013; Wang et al., 2014). Using public algorithms [TargetScan (http://www.targetscan.org/vert_72/) and Diana Tools (http://diana.imis.athena-innovation.gr/DianaTools/index.php)], we identified putative binding sites for five miRNAs-3p within the highly conserved human *YY1* 3′UTR sequence: let-7b-3p, miR-125a-3p, miR-130b-3p, miR-200a-3p and miR-381-3p (Fig. 5B). Of note, let-7b-3p, miR-125a-3p and miR-130b-3p were listed in the panel of miRNAs with higher levels in PPB serum (Murray et al., 2014). We did not observe a significant difference in let-7b-3p expression levels between PPB and control lung specimens (Fig. 5C). In contrast, miR-125a-3p and miR-130b-3p levels were significantly elevated in PPB lung biopsies compared to controls, and the difference (15.0-times) was considerable for miR-125a-3p (*P*adj=0.01; Table S4). The 3.2-times increase in miR-125a-3p levels in CPAM versus control lung samples was not significant (*P*adj>0.99; Fig. 5C). Thus, the miRNA profile detected in PPB serum reflected, in part, the miRNA population prevailing in PPB lung tissue.

**Fig. 6.**
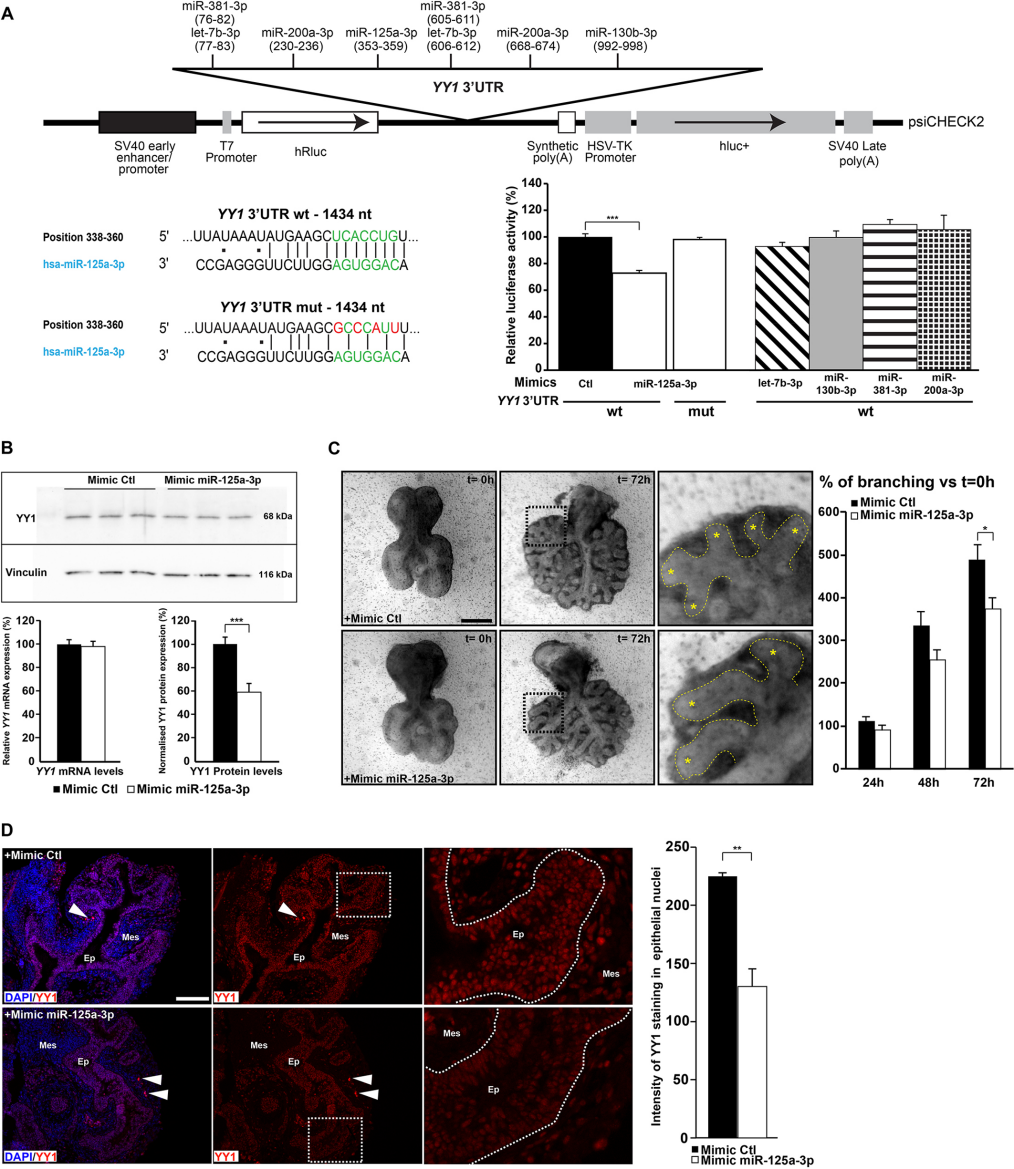
**miR-125a-3p controls YY1 expression.** (A) Diagram of the luciferase reporter used to test miRNA binding sites in HEK293T cells co-transfected with (1) psiCHECK2 vectors carrying *YY1* 3′UTR wild type (wt) or mutated at the predicted miR-125a-3p binding site (mut), and (2) control, miR-125a-3p, let-7b-3p, miR-130-3p, miR-381-3p or miR-200a-3p mimics (top). Only addition of miR-125a-3p mimic reduced luciferase activity levels, which were restored when the miR-125a-3p binding site was mutated (bottom right). The miR-125a-3p seed sequences are indicated in green and the mutation sites are in red (bottom left). (B) Analysis of endogenous *YY1* mRNA (bottom left) and protein (bottom right) expression in A549 lung adenocarcinoma cells transfected with miR-125a-3p mimic by qRT-PCR and western blot assays, respectively. One representative western blot experiment is shown (top). Protein expression levels were calculated by measuring band intensity with ImageJ software, and YY1 expression levels were normalized to vinculin levels. Addition of miR-125a-3p mimic resulted in decreased YY1 protein expression. Samples were tested in triplicate and all experiments were performed two to three times. (C) Lung explants from *Yy1*^flox^;*Dermo1*^+/Cre^ embryos were cultured for 3 days in the presence of control or miR-125a-3p mimics (left). Right column shows higher magnification images of the boxed areas from the corresponding middle column. Yellow asterisks indicate terminal buds; yellow dashed lines outline epithelium. Quantification of the number of terminal buds at 24, 48 and 72 h is represented as the percentage of branching increase versus *t*=0 h (right). (D) Representative micrographs showing YY1 immunostaining in lung explants from *Yy1*^flox/flox^;*Dermo1*^+/Cre^ embryos cultured in the presence of control or miR-125a-3p mimics (left). Right column shows higher magnification images of the boxed areas from the corresponding middle column. Bright red staining indicated by white arrowheads corresponds to autofluorescence from remaining blood cells in lung explants. White dotted lines outline epithelium. Quantification of fluorescence intensity in YY1-positive nuclei was performed with ImageJ software (right). Seven to nine explant cultures were used per condition. Ep, epithelium; Mes, mesenchyme. Means±s.e.m. *P<0.05, **P<0.01, ***P<0.001 (unpaired Student's *t*-test). Scale bars: 400 µm (C), 100 µm (D).

To address the role of miRNAs-3p in YY1 regulation, we co-transfected HEK293T cells with a luciferase translational reporter carrying wild-type *YY1* 3′UTR sequences and synthetic miRNA mimic for each candidate (Fig. 6A). Only addition of the miR-125a-3p mimic significantly reduced luciferase activity by 27% (*P*=0.0005), which was restored to control levels when the miR-125a-3p seed was mutated.

To determine whether the negative regulation of YY1 expression by miR-125a-3p observed in HEK293T cells also occurred in lung epithelial cells, we used, as a cell model, the A549 human lung adenocarcinoma cell line to test endogenous *YY1* expression in response to a miR-125a-3p mimic. Addition of a miR-125a-3p mimic did not impact *YY1* RNA levels, but significantly reduced YY1 protein expression by 41% (*P*=0.0004; Fig. 6B). A similar trend was also observed when the experiments were repeated in Calu-3 cells, another lung adenocarcinoma cell line (not shown). Thus, miR-125a-3p is a strong candidate for post-transcriptional control of YY1 expression in lung cells.

To determine whether miR-125a-3p functionally regulates lung development, lung explants from E11.5 mouse embryos were cultured in the presence of miR-125a-3p or control mimics. To sensitize the system in a way that the effect of miR-125a-3p on *Yy1* epithelial transcripts could be better observed, we used lung explants from *Yy1*^flox^;*Dermo1*^+/Cre^ mutant embryos, in which *Yy1* mesenchymal transcripts are depleted. The specific mutation of *Yy1* function in lung mesenchyme by the Dermo1-Cre recombinase causes collapsed lungs and respiratory failure at birth but does not inhibit branching of lung explant cultures (Bérubé-Simard et al., 2014; not shown). In response to treatment with miR-125a-3p, *Yy1*^flox^;*Dermo1*^+/Cre^ lung explants presented 23% fewer epithelial branches than specimens treated with control mimics after 3 days in culture (*P*=0.03; Fig. 6C). Moreover, they showed a 42% decrease in YY1 protein expression (*P*=0.003) as revealed by YY1 immunofluorescence (IF) staining and measurement of signal intensity with the ImageJ software (Fig. 6D). This supported the concept that a gain in miR-125a-3p in epithelial lung cells is detrimental to branching morphogenesis, and that this action may be mediated, in part, via repression of YY1 protein expression.

### FGF9 misexpression in *Yy1* mutants

miR-140-5p was shown to downregulate *Fgf9* expression in the developing lung but its misexpression in PPB lung specimens was not demonstrated (Yin et al., 2015). We assessed miR-140-5p expression in PPB lung biopsies and found no change in miR-140-5p levels compared to controls (Fig. 5D). This suggested that the augmented *FGF9* expression seen in PPB specimens could not be explained by decreased miR-140-5p expression resulting from *DICER1* missense mutations.

**Fig. 7.**
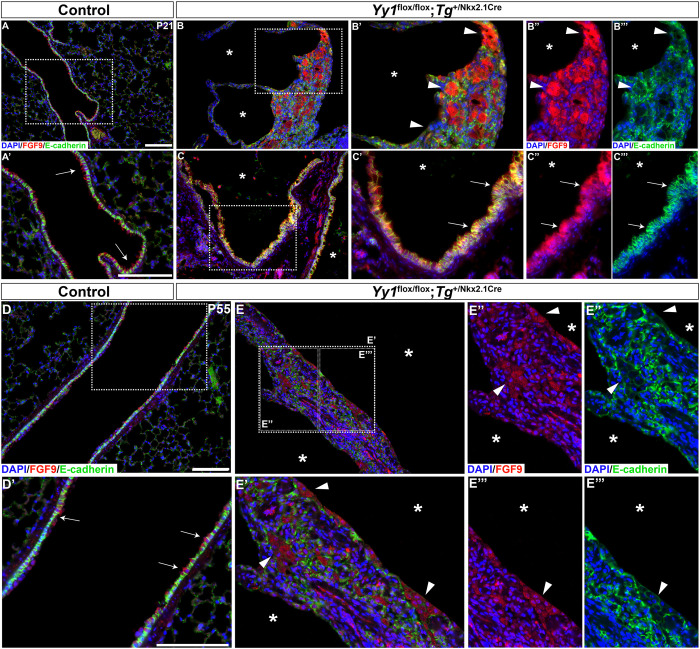
**FGF9 lung epithelial expression is augmented in *Yy1*^flox/flox^;*Tg*^+/Nkx2.1Cre^ mice.** (A-E‴) Representative micrographs showing co-immunostaining of E-cadherin (green) and FGF9 (red) in control and *Yy1*^flox/flox^;*Tg*^+/Nkx2.1Cre^ mice at postnatal day (P)21 (A-C‴) and P55 (D-E‴). FGF9 epithelial expression (arrows) was increased in the cystic areas (asterisks) of the lungs of mutants. Tissue disorganization (arrowheads) in cystic walls was observed. Boxed areas are shown at higher magnification in the corresponding images below or right. Scale bars: 100 µm.

We previously reported that *Fgf9* RNA levels were diminished in lungs from *Yy1*^flox/flox^;*Shh*^+/Cre^ embryos (Boucherat et al., 2015). *Fgf9* is expressed in lung epithelium and mesothelium (Yin et al., 2011), raising the concern that local changes in specific lung cell populations might not be detectable by a global qRT-PCR approach. To address tissue specificity of FGF9 expression in *Yy1* mutants, we performed FGF9 immunostaining on lung sections. The neonatal lethality of *Yy1*^flox/flox^;*Shh*^+/Cre^ mice precludes their study after birth. However, lung epithelial deletion of *Yy1* function with the *Tg*^+/Nkx2.1Cre^ mice causes a less-severe cystic phenotype. The Nkx2.1-Cre recombinase directs Cre activity in the proximal respiratory epithelium, and ablation of *Yy1* function with the Nkx2.1-Cre produces cysts in the proximal region of the lobes, which allows the survival of few mutants (Boucherat et al., 2015). Furthermore, lungs from *Yy1*^flox/flox^;*Tg*^+/Nkx2.1Cre^ survivors present high proliferation, a characteristic of an evolving PPB-like phenotype (Boucherat et al., 2015). When we looked at FGF9 protein expression in lungs from *Yy1*^flox/flox^;*Tg*^+/Nkx2.1Cre^ mutant mice, we observed an increased expression in the epithelium of the proximal lung region at postnatal ages (Fig. 7). Co-labeling with E-cadherin also revealed a profound disorganization of lung tissues, with epithelial cells intermingled in the mesenchyme (Fig. 7B,E). Taken together, these data indicated that the inactivation of *Yy1* in lung epithelium promotes FGF9 epithelial expression.

## DISCUSSION

### A distinctive molecular signature for PPB

PPB is a poorly understood developmental disorder that can progress to an aggressive intrathoracic pediatric malignancy. The diagnosis of this very rare pathology is challenging. Once detected, young patients are submitted to multi-modal therapies, including surgery and chemotherapy, to reduce recurrences. PPB is associated with *DICER1* loss-of-function and missense mutations resulting in an abnormal quantitative and qualitative miRNA panel that can perturb target gene expression. However, the downstream molecular events that participate in PPB occurrence remain undefined. In addition, there has been a paucity of molecular markers that could facilitate diagnosis. There is thus a need to identify the mechanisms and key molecules underlying PPB pathogenesis for improvement of early diagnosis and development of less-invasive treatments.

The specific inactivation of *Yy1* in mouse lung epithelium provides a valuable model to study the molecular mechanisms associated with the abnormal formation of epithelial lung cysts, a feature of early stages of PPB. Several genes differentially expressed in lungs from *Yy1* mutant mouse embryos showed similar variations in PPB lung biopsies that were not found in CPAM patients. For the *FGF9*, *FGF10*, *SHH* and *FGFR2-IIIB* genes, the changes in expression observed in PPB samples were previously reported (Lezmi et al., 2013; Yin et al., 2015). Moreover, a transcriptomic approach was recently applied to CPAM lung epithelium specimens (Lezmi et al., 2020). Candidate epithelial genes we found unaffected in CPAM patients also showed no expression differences in their transcriptome analysis. Thus, despite variations among human samples, correlation with other studies validates our method.

The PPB molecular signature includes changes in expression of the *ETV4*, *ETV5*, *ELF5*, *IRX2* and *IRX5* genes, all coding for lung epithelial transcription factors. Knockdown of Irx genes in rodents reduces lung branching and causes tubule distension resembling lung cysts (van Tuyl et al., 2006). Similarly, Etv ablation in murine lung epithelium leads to less branching and dilation of branch tips, while *Elf5* downregulation is associated with abnormal branching (Metzger et al., 2007; Herriges et al., 2015). Thus, the reduction in *Etv5*, *Elf5*, *Irx2* and *Irx5* expression levels in *Yy1* mouse mutants and PPB patients is coherent with the observed lung cystic phenotype. It also raises questions about the potential role of YY1 as a positive transcriptional regulator governing the expression of these different players. In contrast, the increased *Etv4* expression remains unexplained, especially because YY1 was shown to activate *Etv4* gene expression by mediating enhancer-promoter interactions at the *Etv4* locus (Weintraub et al., 2017). *Etv4* and *Etv5* are considered bona fide targets of FGF10 signaling in the developing lung (Jones et al., 2019). However, their inverse variation in expression in PPB human patients and *Yy1* mouse embryonic lung specimens indicated that other molecular mechanisms differentially regulate *Etv4* and *Etv5* expression, and suggested that they might respond to distinct signals and have specific functions still to be defined.

**Fig. 8.**
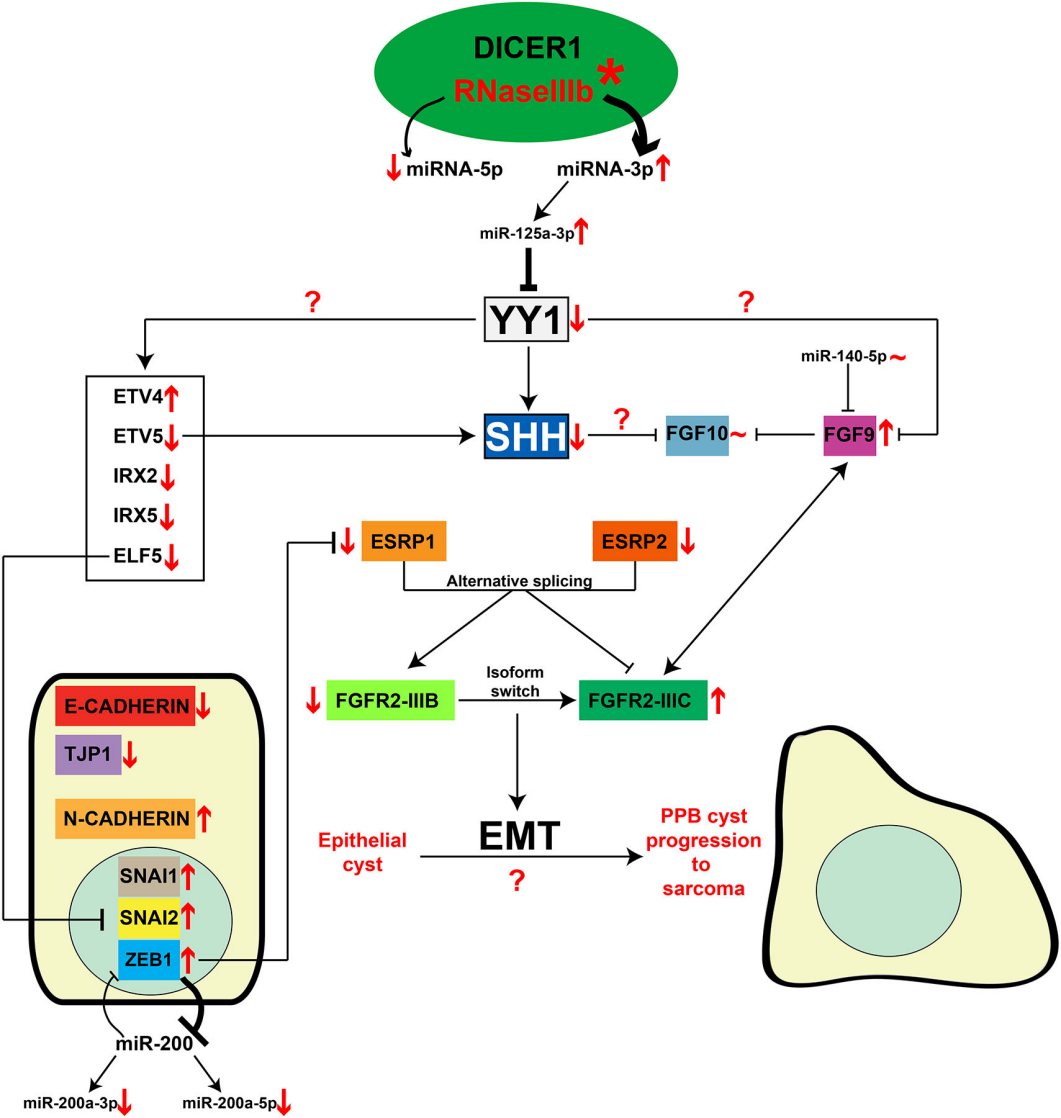
**Model for the involvement of YY1 in PPB pathogenesis.** In the PPB context (text and arrows in red), the somatic missense mutation (asterisk) in the RNaseIIIb domain of the DICER1 protein modifies the miRNA profile, causing a bias toward the generation of miRNAs-3p over miRNAs-5p. Higher miR-125a-3p levels are found in PPB, and they bind to *YY1* 3′UTR to inhibit YY1 protein expression. Reduced YY1 expression correlates with altered expression levels of several lung epithelial transcription factors – *ETV4*, *ETV5*, *ELF5*, *IRX2* and *IRX5* – as previously observed in *Yy1*^flox/flox^;*Shh*^+/Cre^ mutant mice. Accordingly, *SHH*, a direct transcriptional target of both YY1 and ETV5, shows reduced expression in PPB lung tissues. Decreased expression of epithelium-specific genes, like *CDH1*, *TJP1* and the *FGFR2-IIIB* isoform, is also observed in PPB samples. In parallel, expression of mesenchyme-specific markers – such as *SNAI1*, *SNAI2*, *ZEB1*, *CDH2* and the *FGFR2-IIIC* isoform – is increased. The upregulation of ZEB1 may be explained by the reduced expression of miR-200a in PPB. High ZEB1 expression levels are known to inhibit ESRP1. Together, the data suggest that EMT is promoted in PPB and may be involved in the progression of PPB epithelial cysts toward sarcoma. Expression of the splicing regulatory factors *ESRP1* and *ESRP2* is reduced, likely contributing to the FGFR2 isoform switch from IIIB to IIIC observed in PPB patients. The FGFR2-IIIC receptor preferentially binds the FGF9 ligand, which is highly expressed in PPB samples and *Yy1* mutant mice. This suggests that YY1 participates in the regulation of *FGF9* expression. There is no clear evidence that miR-140-5p, a previously identified post-transcriptional regulator of FGF9, is associated with increased *FGF9* expression in PPB samples. No change in *FGF10* expression levels is seen in PPB lung samples in contrast to the dramatic increase previously observed in the *Yy1*^flox/flox^;*Shh*^+/Cre^ mouse model. In human lung development, the exact role of FGF10 remains undefined. However, one possible explanation for the unchanged *FGF10* levels in PPB is that FGF9, known to inhibit *FGF10* expression in human lung, could act to maintain stable *FGF10* levels. Thus, YY1 occupies a central position in the mechanism of PPB pathogenesis, as a downstream target of the abnormal epithelial DICER1-cleaved miRNA profile as well as a transcriptional regulator of key molecules of lung development. The exact role of YY1 in the observed changes of epithelial gene expression in PPB remains to be determined.

Reduced expression of the SHH signaling molecule was observed in PPB but not in CPAM lung specimens, a coherent result because *Shh* is a direct transcriptional target of YY1. *Shh* was also shown to be under the direct positive transcriptional control of ETV5, which participates in the FGF10–SHH feedback loop that governs lung branching (Herriges et al., 2015). Thus, the combined reduction in YY1 and ETV5 expression in PPB patients likely explains the decreased *SHH* expression. It also suggests that the downregulation of SHH is a critical convergence point of the molecular pathways that underlie PPB pathogenesis (Fig. 8).

The FGF10–SHH feedback loop is central for murine lung branching morphogenesis. Surprisingly, in PPB patients, decreased expression of SHH was not accompanied by augmented *FGF10* expression, as seen in *Yy1* mouse mutant embryos. In the mouse lung, FGF10 produced by clustered mesenchymal cells drives the growth of endodermal buds during the branching process (Bellusci et al., 1997). Addition of exogenous FGF10 to mouse lung explants induces branch formation. The FGF10 branch-promoting effect is counteracted by SHH secreted by the lung epithelium, which signals to the mesenchyme to inhibit FGF10 expression. In humans, FGF10 expression is diffuse throughout the lung parenchyma. When added to human lung explants, FGF10 inhibits branching but favors cyst formation (Danopulos et al., 2019). According to these data, FGF10 seems dispensable for the growth of distal epithelial tips in humans, which may reconcile the morphological phenotype with the absence of significant difference in *FGF10* expression in PPB versus control lung biopsies (Nikolić et al., 2017; Danopulos et al., 2019). The SHH expression profile is highly similar in developing human and mouse lungs, and only slight differences were reported in the expression of SHH receptors and signaling effectors, which together support the notion that activity of the SHH pathway is conserved in human lung development compared to vertebrate models (Zhang et al., 2010). However, the exact role of SHH in relation to FGF10 and human lung branching morphogenesis remains an open question (Fig. 8).

Timing variations could contribute to the different behaviors of *FGF10* expression in PPB patients versus *Yy1* mouse mutants. Increased *Fgf10* expression was detected in *Yy1* mutant lungs at the pseudoglandular stage, while the human biopsies used for evaluating gene expression were obtained from young children, implying that their lungs were at the alveolar stage and beyond. We measured *Fgf10* expression in lungs from *Yy1*^flox/flox^;*Tg*^+/Nkx2.1Cre^ survivors at postnatal ages corresponding to the alveogenesis period and later (Fig. S1). No change in *Fgf10* expression was found, a result comparable to the lack of difference in *FGF10* expression in PPB patients. This demonstrates the importance of studying comparable developmental stages. It would be informative to determine whether *FGF10* expression levels are increased in lungs from PPB human embryos at the pseudoglandular stage (from 5 to 17 weeks of gestation).

Another possibility that could participate in the difference in *FGF10* expression levels between PPB patients and *Yy1* mouse mutants is FGF9. FGF9 was shown to inhibit *FGF10* expression in human lung explants, whereas the opposite was observed in mouse lung cultures (del Moral et al., 2006; Danopulos et al., 2019). The high *FGF9* expression seen in PPB lungs might inhibit *FGF10* expression, neutralizing the *FGF10* increase resulting from repressed *SHH* expression and thus contributing to maintenance of stable *FGF10* levels (Fig. 8). These different explanations are not mutually exclusive and they raise questions about the exact contribution of FGF10 to human lung development.

FGF9 is highly expressed in lungs from PPB patients (Fig. 1; Yin et al., 2015). In mice, the epithelial deletion of *Dicer1* causes *Fgf9* overexpression in lung epithelium, which was shown to mediate the cystic lung phenotype observed in *Dicer1* mutants. In humans, FGF9 induces cyst formation in lung explants, similar to its action in mice (Danopulos et al., 2019). The impact of the *Dicer1* mutation on *Fgf9* expression establishes that FGF9 is under the regulation of miRNAs, and one candidate is miR-140-5p (Yin et al., 2015). However, decreased miR-140-5p levels expected from the *DICER1* missense mutation were not observed in PPB lung biopsies, implying that changes in miR-140-5p expression are not the cause of the augmented *FGF9* expression in PPB specimens. Other miRNAs are known to target FGF9 expression in lung cells but their role in PPB has yet to be established (Rao et al., 2019). Moreover, the increased FGF9 protein expression detected in lungs from *Yy1*^flox/flox^;*Tg*^+/Nkx2.1Cre^ mutant mice raised the possibility that regulatory mechanisms involving YY1 might contribute to control FGF9 lung expression (Fig. 8). Finally, increased FGF9 expression levels were reported in lung epithelium from CPAM fetal specimens, suggesting that high expression of FGF9 might be a prerequisite for epithelial cyst formation in a DICER1-independent manner (Jancelewicz et al., 2008).

### A role for EMT in PPB progression

The function of FGF receptors appears to be conserved between humans and mice (Danopulos et al., 2019). The significant decrease in *FGFR2-IIIB* expression in PPB lung specimens correlated with reduced activity of the signaling pathway shown by the diminished expression of FGF10 effectors. Combined with the concomitant increased expression of the *FGFR2-IIIC* oncogenic isoform, deregulation of FGF signaling in PPB specimens might unbalance epithelial-mesenchymal homeostasis and stimulate cancer progression, as demonstrated in other cancers (Fig. 8; Oltean et al., 2006; Zhao et al., 2013; Ranieri et al., 2016). This is further supported by the observation that EMT transcriptional drivers, such as SNAI1, SNAI2 and ZEB1, were found to be highly expressed in PPB lung specimens, promoting the activation of mesenchymal proteins and the downregulation of epithelial markers such as E-cadherin and TJP1.

Interestingly, several of the genes showing expression changes in PPB were previously linked together and associated with EMT and cancer. For example, in lung cancer cells, ZEB1 was shown to downregulate *ESRP1* expression through direct binding to its promoter, contributing to a positive loop fostering EMT (Roche et al., 2013). In breast cancer cells, ELF5 transcriptionally represses *SNAI2* expression (Chakrabarti et al., 2012). The negative correlation between *ELF5* and *SNAI2* expression seen in breast tumor clinical samples is similar to that observed in PPB specimens, suggesting that the decreased expression of *ELF5* in PPB lung specimens can promote EMT. Finally, ZEB proteins and miRNA-200 family members form a double-negative feedback loop known to regulate EMT in tumorigenesis (Burk et al., 2008). In PPB, the decreased expression of miRNAs from the miR-200 family negatively correlates with the higher expression of ZEB1 protein, which supports the notion that it can stimulate EMT (Fig. 8).

*Fgfr2-IIIb*, *Esrp2* and *Cdh1* genes all showed significantly decreased expression in *Yy1*^flox/flox^;*Shh*^+/Cre^ mutant samples, raising questions about a potential role for YY1 in the control of EMT. Most of the changes in expression of EMT players, such as *Fgfr2-IIIc*, *Esrp1*, *Snai1*, *Snai2*, *Tjp1*, *Cdh2* and *Zeb1*, were detected in PPB specimens but not in the *Yy1* mouse model, indicating that if YY1 is involved, its regulatory role is modest, with limited impact on EMT. However, *Yy1*^flox/flox^;*Shh*^+/Cre^ mutants only model early stages of the PPB disease due to their death at birth. EMT is a gradual process, with several cellular states presenting intermediate transcriptional characteristics (Pastushenko and Blanpain, 2019). Therefore, gene expression modulation might extend over a period encompassing early to advanced stages of PPB progression, the latter not being reproduced in *Yy1* mutants.

So far, little is known about how PPB evolves. Primitive small mesenchymal cells residing underneath the benign epithelial cyst surface are described as a histopathologic feature of Type I PPB (Hill et al., 2008). Their overgrowth is proposed to generate a cystic and solid neoplasm (Type II PPB) and eventually a purely solid high-grade sarcoma (Type III PPB). This is supported by the increased proliferative index observed in the mesenchyme compartment of PPB lung tumors (Fig. S2; Hill et al., 2008). However, the global deregulation of genes related to EMT programs in PPB specimens suggested that EMT might participate in the progression of a purely epithelial cystic lesion to a solid sarcomatous tumor. The design of a mouse model able to closely recapitulate PPB through its characteristic tumor pathological changes is therefore necessary to explore the role of EMT in the mechanisms of PPB pathogenesis.

### YY1, a downstream target of the abnormal epithelial DICER1-cleaved miRNA profile

miR-125a-3p was found to post-transcriptionally repress YY1 expression in HEK293T cells as well as in lung epithelial cell lines. Moreover, addition of miR-125a-3p to cultured lung explants decreased their potential to correctly branch, indicating that miR-125a-3p overexpression is harmful for lung development. The phenotype of miR-125a-3p-treated lung explants was not as severe as the one seen in *Yy1*^flox/flox^;*Shh*^+/Cre^ mutant embryos, the latter corresponding to a full deletion of *Yy1* function in lung epithelium. This indicated that miRNA-mediated post-transcriptional repression is insufficient to completely abolish YY1 activity. Nonetheless, the concordance between the defective branching phenotypes in *Yy1*^flox/flox^;*Shh*^+/Cre^ mutants and lung explants cultured with miR-125a-3p agrees with the notion that YY1 is a downstream target of the abnormal epithelial DICER1-cleaved miRNA profile (Fig. 8). However, we cannot rule out the possibility that miR-125a-3p may also act on other regulators of lung branching that contribute to the PPB phenotype.

miR-125a-3p levels were previously reported to be abnormally elevated in serum from a PPB patient, and the same result was observed in lung biopsies from PPB patients (Fig. 5; Murray et al., 2014). This establishes that miRNA changes in PPB serum reflect the miRNA status in PPB lung, and raises the possibility that measuring miR-125a-3p serum levels could be envisaged as a PPB diagnostic tool. It must be underscored that upregulated miR-125a-3p expression was recently reported at early stages of lung adenocarcinoma, suggesting that increased levels of miR-125a-3p may also be detrimental in adult lung cancer (Zeybek et al., 2019).

In conclusion, we present evidence for a unique PPB molecular signature that distinguishes PPB from CPAM and could thus contribute to improved diagnostic methods and to better early management of the disease. This signature also closely matches the molecular changes seen in *Yy1* mutants. This confirms the utility of the conditional ablation of *Yy1* in the developing lung epithelium as a model for the early stage of PPB, and to decipher the molecular mechanisms leading to the abnormal formation of epithelial lung cysts. These findings also endorse the hypothesis that reduced expression of YY1, consequent to the abnormal miRNA profile, contributes to PPB pathogenesis via its impact on the expression of key regulators of lung development.

## MATERIALS AND METHODS

### Human tissue

This study was conducted using anonymized specimens from control, CPAM and PPB patients from the Department of Anatomo-Pathology of Hôpital Necker-Enfants Malades (Paris, France), from McGill University/Segal Cancer Centre (Montréal, Canada) and from the International Pleuropulmonary Blastoma Registry (Minneapolis, MN, USA). Informed consent was obtained from all subjects, and all clinical investigation was conducted according to the principles expressed in the Declaration of Helsinki. The procedures were approved by the respective institutional ethics committee and by the CHU de Québec-Université Laval research ethics committee (project MP-20-2020-4809). When possible, the sex of specimens was determined by qRT-PCR expression of the Y-specific lung-expressed gene, *UTY* (Greenfield et al., 1996). Characteristics of patients are summarized in Table S2.

### Mice, genotyping and tissue collection

*Yy1*^flox/flox^ mice were obtained from Dr Shi (Affar et al., 2006), and the *Shh*^+/Cre^ [*Shh*^tm1(EGFP/cre^^)Cjt^] and *Tg*^+/Nkx2.1Cre^ [Tg^(Nkx2-1-cre)2Sand^] deleter strains were purchased from The Jackson Laboratory (Harfe et al., 2004; Xu et al., 2008). The *Dermo1*^+/Cre^ [*Twist2*^tm1(Cre)Dor^] line was obtained from Dr Ornitz (Yu et al., 2003). Only individuals carrying the *Yy1*^flox/flox^;*Shh*^+/Cre^ and *Yy1*^flox/flox^;*Tg*^+/Nkx2.1Cre^ genotypes presented cystic defects, and *Yy1*^+/flox^;*Shh*^+/Cre^ and *Yy1*^+/flox^;*Tg*^+/Nkx2.1Cre^ genotypes were used as controls. Age of the embryos was estimated by considering the morning of the day of the vaginal plug as E0.5. Experimental specimens were genotyped by PCR analyses. For RNA extraction, lungs were snap-frozen in liquid N_2_. Experiments were performed according to the guidelines of the Canadian Council on Animal Care and approved by the institutional animal care committee.

### Cell lines

Cell lines were obtained from American Type Culture Collection. Human embryonic kidney cells (HEK293T) were maintained in Dulbecco's modified Eagle medium (DMEM) supplemented with 10% fetal bovine serum (FBS) and antibiotics. A549 and Calu-3 cells, two human hypotriploid lung adenocarcinoma cell lines that express *YY1* and miR-125a-3p, were cultivated in F12K medium (Corning) and DMEM (Gibco), respectively, containing 10% FBS and antibiotics.

### Luciferase plasmids

The *YY1* 3′UTR region was obtained from genomic DNA of GM12878 human lymphocyte cell line by PCR amplification with the following primers: F, 5′-CTCGAGCCTATGTGTGCCCCTTCGAT-3′ and R, 5′-CGCCGGCGTGCACCCAGTGTCCATTTAGAA-3′. The 1567 bp PCR product was cloned into psiCHECK2 vector (Promega). Mutations in miR-125a-3p binding sites were obtained by directed PCR mutagenesis with the following primers: F, 5′-GTAAGCAAAATGGGCGCTTCATATTTATAAG-3′ and R, 5′-AATTTTTTTAATTTTGTATTTTCCAAGTGTGC-3′.

### Cell transfection and luciferase reporter assay

HEK293T cells were transfected with the X-tremeGENE siRNA transfection reagent (Roche) with 300 ng plasmid DNA and 1 µl of 20 µM mimics (Dharmacon) in 50 µl medium. Forty-eight hours post-transfection, cells were collected, and Renilla and Firefly luciferase activities were assessed with the Dual-Glo luciferase assay system (Promega) according to the manufacturer's instructions. Luminescence readings were acquired using a Luminoskan Microplate Luminometer (Thermo Fisher Scientific). The Renilla luciferase signal was normalized to the Firefly luciferase activity to adjust for variations in transfection efficiency. Samples were tested in triplicate and all experiments were performed at least two times.

A549 and Calu-3 cells were plated into 24-well plates and transfected with 1 µl of 20 µM miR-125a-3p or miRIDIAN miRNA mimic transfection control with Dy547 (Dharmacon) with Lipofectamine-3000 (Invitrogen), according to the manufacturer's specifications. Cells were collected after 48 h and processed for RNA extraction with TRIzol™ (Invitrogen), according to the manufacturer's instructions, for qRT-PCR assays or kept in protein lysis buffer for western blot analysis (Tabariès et al., 2007). Samples were tested in triplicate and all experiments were performed at least three times.

### Histology, IHC and IF analyses

Experiments were performed as described (Boucherat et al., 2014). The following primary antibodies were used: anti-E-cadherin (clone 36/E-cadherin 610181, BD Biosciences; 1/5000 for human sections, 1/200 for mouse sections), anti-FGF9 (ab71395, Abcam; 1/50), anti-Ki67 (also known as Mki67; NCL-L-Ki67-MM1, Leica Biosystems; 1/200), anti-YY1 (ab109237, Abcam; 1/1000) and anti-ZEB1 (ab203829, Abcam; 1/50). The biotinylated secondary antibodies used for IHC experiments were as follows: goat anti-rabbit antibody (BA-1000, Vector Laboratories; 1/500), goat anti-mouse antibody (115-065-003, Cedarlane; 1/500) and swine anti-goat antibody (CLCC50015, Cedarlane; 1/250). Immunostaining was revealed with Liquid DAB^+^ Substrate Chromogen System Kit (3,3′-diaminobenzidine; DAKO). The fluorescent antibodies used for IF experiments were as follows: goat anti-mouse Alexa Fluor 488 (A21121, Invitrogen; 1/250), goat anti-rabbit Alexa Fluor 555 (A21428, Invitrogen, 1/250), goat anti-rabbit Alexa Fluor 647 (A21245, Invitrogen, 1/250) and donkey anti-rabbit Cy5 (711-175-152, Jackson ImmunoResearch Laboratories; 1/200). For IHC and IF experiments, nuclei were visualized by Hematoxylin and 4′,6-diamidino-2-phenylindole (DAPI) staining, respectively.

Measurement of YY1 IF staining was obtained by quantification of gray levels in epithelial nuclei using the ImageJ software (https://imagej.nih.gov/ij/). The procedure was adapted from Crowe and Yue (2019). Briefly, RGB multi-channel pictures were converted into 16-bit grayscale images. Nuclei were highlighted by setting a minimal threshold and then converting into a binary image. The watershed option was applied to delineate nuclei not clearly separated. Automatic detection of nuclei was achieved with the ‘analyze particles’ option in ImageJ, which generates regions of interest (ROIs) corresponding to each nucleus. Single-channel images corresponding to YY1 fluorescence signal were converted into binary images, from which pixel intensities were quantified from 0 to 255. Results are presented as means±s.e.m. The means were obtained from at least two to three sections per specimen from two to five specimens per genotype. The number of nuclei analyzed ranged from 750 to 2000 per section.

Quantification of E-cadherin and ZEB1 IHC staining intensities was performed with the QuPath open source software for digital pathology image analysis (Bankhead et al., 2017; https://qupath.github.io). Brightfield images of Hematoxylin and DAB staining were first processed with the built-in visual stain editor with default options to estimate stain channels, which helps to improve stain separation in brightfield images. QuPath then uses the color deconvolution method for stain separation (Ruifrok and Johnston, 2001). A cell-detection command was applied to the Hematoxylin channel to clearly detect all nuclei using QuPath integrated segmentation algorithms. Detection and quantification of DAB^+^ cells were obtained using the object classification options. Intensity for each epithelial and/or mesenchymal cell was then extracted from QuPath for analysis. Values were obtained from five to seven human specimens per condition. On average, 465 positive cells per specimen were analyzed.

### qRT-PCR experiments

Lung total RNA was isolated from individual E15.5 mouse embryos and treated with DNaseI (Invitrogen). qRT-PCR experiments on mouse RNAs were performed as described (Boucherat et al., 2012). Five specimens were used per genotype tested.

Human specimens were obtained fresh frozen or embedded in optimal cutting temperature (OCT) compound. RNA extraction from fresh frozen specimens was performed as described (Boucherat et al., 2012). Samples in OCT were cut in 20-µm-thick sections, and 20-30 sections per specimen were used for extraction with TRIzol™ (Invitrogen), according to the manufacturer's instructions. cDNA was synthetized with Superscript™ IV Reverse transcriptase (Invitrogen) using 1 µg total RNA and random primers. qRT-PCR was performed with Power Sybr Green PCR Master Mix (Invitrogen) and a thermal cycler ABI PRISM 7000. All primer sequences are listed in Table S3. Reference gene (GOR) stability and optimal number of GORs were determined using geNorm algorithm module in qbase+ software, version 3.0 (Biogazelle, Zwijnaarde, Belgium; www.qbaseplus.com). Primer sets were selected such that qRT-PCR amplification efficiencies obtained from standard curves of serial dilutions spanning at least five orders of magnitude showed real-time efficiency rates ranging from 1.90 to 2.10 with high linearity (Pearson correlation coefficient *r*>0.98). For each gene of interest (GOI) tested, the level of gene expression was normalized to the geometric mean of the expression levels of the two most stable GORs with low variability among the samples (<0.15), here *RPL19* and *HPRT1*, and calculated according to the Pfaffl equation (Pfaffl, 2001; Vandesompele et al., 2002):

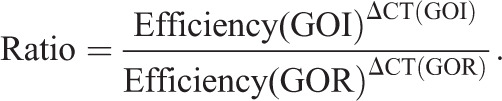


Seven to 16 specimens were used per genotype for gene expression analysis with human tissues. miRNA expression analysis was performed according to TaqMan Small RNA Assays protocol from Applied Biosystems (Life Technologies). Briefly, 10 ng of total RNA was reverse transcribed with TaqMan MicroRNA Reverse Transcription kit using specific primers (Life Technologies). qRT-PCR experiments were carried out with Universal PCR Master Mix and specific probes using an ABI PRISM 7700 detection system. Quantifications of miR-125a-3p (Invitrogen, Assay ID 002199), miR-200a-3p (Assay ID 000502), miR-200a-5p (Assay ID 001011) and miR-140-5p (Assay ID 001187) were normalized to U6 snRNA (Assay ID 001973). Three to 14 specimens were used per genotype tested for gene expression analysis with human tissues.

### Western blotting

Equal amounts of total proteins per well were separated by SDS-PAGE. Gels were transferred onto nitrocellulose membranes and incubated with the following primary antibodies: anti-YY1 (SC-1703X, Santa Cruz Biotechnology; 1/2000) and anti-vinculin (clone hVIN-1, Sigma-Aldrich; 1/2000). Vinculin was used as loading control. Antigen-antibody complexes were detected with an anti-IgG antibody coupled to horseradish peroxidase and revealed using an enhanced chemiluminescence kit (Azure Biosystems). Quantification of YY1- and vinculin-immunoreactive bands was obtained by densitometry analyses with the Fluo-S MAX MultiImager-captured images using ImageJ software. YY1 expression levels were normalized to vinculin levels. Samples were tested in triplicate and experiments were repeated four times.

### Lung explant cultures

E11.5 embryonic lungs were dissected and cultured as described (Boucherat et al., 2014). Then, 100 nM miR-125a-3p or miRIDIAN mimic control with Dy547 (Dharmacon) was added with Lipofectamine-3000 (Invitrogen) to lung explants at day 0, according to the manufacturer's protocol. Lung explants were kept for 72 h in serum-free DMEM-F12 medium (Gibco) and collected for immunostaining assay. Seven to nine explant cultures were used per condition.

### Statistical analyses

qRT-PCR data are expressed as relative gene expression levels. Values were normalized to the mean expression of the control group, which was arbitrarily set to 100% expression. Mean±s.e.m. for each group is indicated for each analysis. To establish whether differences between control (*Yy1*^+/flox^*;Shh*^+/Cre^) and mutant (*Yy1*^flox/flox^*;Shh*^+/Cre^) specimens were significant, non-parametric Mann–Whitney test was performed. For the experiments with control, CPAM and PPB biopsies, we used Kruskal–Wallis test followed by Dunn's post hoc comparisons. Bonferroni's correction was used to adjust the *P*-value (*P*adj) for repeated testing.

For Taqman qRT-PCR experiments, the significant differences in expression levels between control and PPB patient specimens were assessed by a non-parametric Mann–Whitney test for the data obtained for miR-200a-3p, miR-200a-5p, miR-130b-3p, let-7b-3p and miR-140-5p. For miR-125a-3p expression analysis in control, PPB and CPAM patients, Kruskal–Wallis test followed by Dunn's post hoc comparisons and Bonferroni's correction were applied.

Student's *t*-test was performed for comparative studies of relative luciferase activity, lung explant culture experiments, endogenous *YY1* mRNA and YY1 protein expression levels in lung cell lines, and IHC and IF staining intensity quantification (Table S4).

Means, s.e.m. and *P*-values for each experiment are listed in Table S4. A significance level inferior to 5% (*P*<0.05 and *P*adj<0.05) was considered statistically significant. Statistical analyses were performed using the Statistical Package for the Social Science (SPSS) computer software version 26.0 (IBM SPSS Statistics, Chicago, IL, USA). Graphs were generated using Prism 8 version 8.2.0 (GraphPad Software, Inc.).

Considering the limited size of the cohorts, we could not statistically test the effect of the sex of the patients in relation to the disease (interaction effect), and the effect of the histotype of the disease for all tested genes. No clear trend supported a sex or a disease type effect on gene expression (Figs 1–3 and 5).

## Supplementary Material

Supplementary information
