# Supplementary material for: Deletion of Yy1 in mouse lung epithelium unveils molecular mechanisms governing pleuropulmonary blastoma pathogenesis
Source: Dis Model Mech. 2020 Dec 29;13(12):dmm045989. doi: 10.1242/dmm.045989 (PMC7790197; doi:10.1242/dmm.045989)
Supplement: Supplementary information [file dmm-13-045989-s1.pdf]

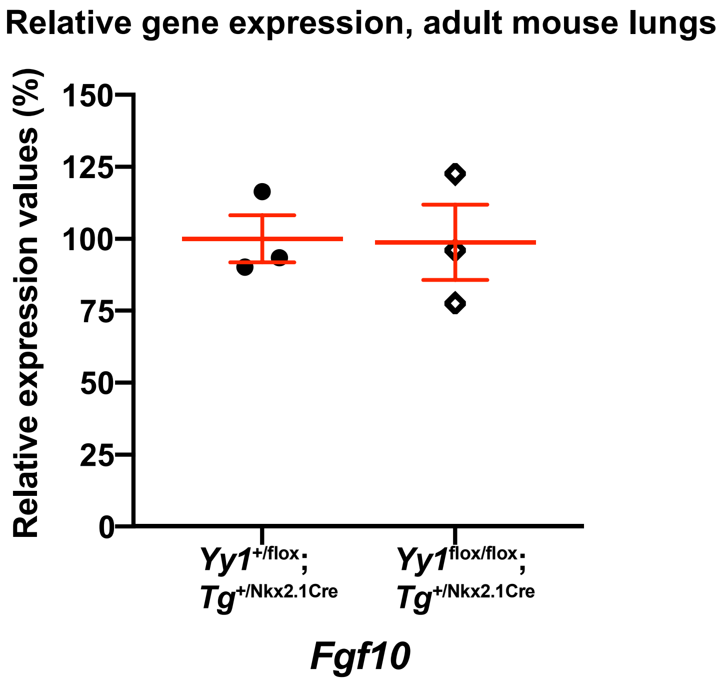

Figure S1. *Fgf10* expression in mouse lung tissues. qRT-PCR analysis of *Fgf10* expression levels in lungs from adult controls and *Yy1*$^{flox/flox}$;*Tg*$^{+/Nkx2.1Cre}$ surviving mice. Mean±s.e.m. are shown. (Mann-Whitney).

Disease Models & Mechanisms • Supplementary information

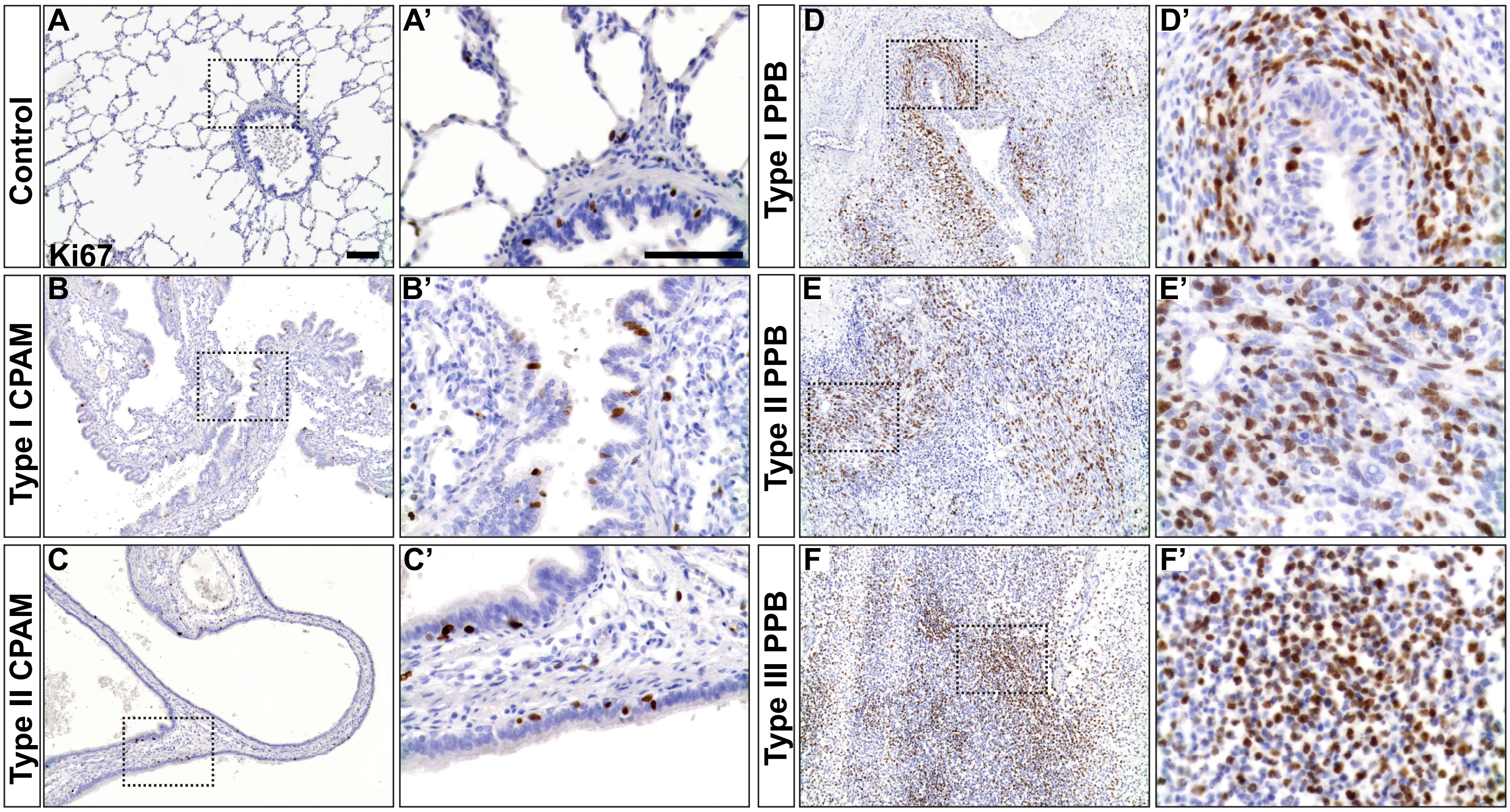

Figure S2. Increased proliferation in PPB lung tissues. (**A-F'**) Representative micrographs showing Ki67 immunostaining in human lung biopsies from controls, CPAM (Type I and Type II) and PPB (Type I, Type II and Type III) patients indicated higher proliferation in the mesenchyme of PPB lung specimens while few proliferating cells were present in controls or CPAM samples. This higher proliferative index was indicative of PPB tumor progression. Right panels show higher magnification of the boxed area from the corresponding left panels. Scale bars: 100µm.

Table S1. Gene expression analyses in lung specimens from *Yy1*$^{flox/flox}$;*Shh*$^{+/Cre}$ mouse mutants and from PPB and CPAM patients

Click here to Download Table S1

Table S2. General characteristics of patients

Click here to Download Table S2

Table S3. List of primer sequences

Click here to Download Table S3

Table S4. Statistical analyses

Click here to Download Table S4
